# Ca^2+^/Calmodulin and Presynaptic Short-Term Plasticity

**DOI:** 10.5402/2011/919043

**Published:** 2011-06-23

**Authors:** Sumiko Mochida

**Affiliations:** Department of Physiology, Tokyo Medical University, 1-1 Shinjuku-6-chome, Shinjuku-ku, Tokyo 160-8402, Japan

## Abstract

Synaptic efficacy is remodeled by neuronal firing activity at the presynaptic terminal. Presynaptic activity-dependent changes in transmitter release induce postsynaptic plasticity, including morphological change in spine, gene transcription, and protein synthesis and trafficking. The presynaptic transmitter release is triggered and regulated by Ca^2+^, which enters through voltage-gated Ca^2+^ (Ca_V_) channels and diffuses into the presynaptic terminal accompanying action potential firings. Residual Ca^2+^ is sensed by Ca^2+^-binding proteins, among other potential actions, it mediates time- and space-dependent synaptic facilitation and depression *via* effects on Ca_V_2 channel gating and vesicle replenishment in the readily releasable pool (RRP). Calmodulin, a Ca^2+^-sensor protein with an EF-hand motif that binds Ca^2+^, interacts with Ca_V_2 channels and autoreceptors in modulation of SNARE-mediated exocytosis.

## 1. Introduction

For memory formation in a neuronal circuit, the primary function of presynaptic terminals is the firing activity-dependent release of neurotransmitters and subsequent recycling of their carrier synaptic vesicles, processes which critically depend on ATP and Ca^2+^. Presynaptic firing of action potentials activates voltage-gated Ca^2+^  (Ca_V_) channels, and Ca^2+^ entry initiates release of neurotransmitters. Ca^2+^  dependence on fast neurotransmitter release is thought to be conferred by the synaptotagmin, a family of Ca^2+^ sensors that interact with SNAREs [1]. Synaptotagmin 1 and 2 are synaptic vesicle proteins with tandem C2 domains that bind Ca^2+^ and ensure the synchronization of Ca^2+^-dependent exocytosis with the presynaptic action potential [2–5]. Neuronal firing activity also controls other protein functions and dynamically remodels synaptic efficacy. Ca^2+^-binding proteins sensing residual Ca^2+^, which accumulates locally in the presynaptic terminal during trains of action potentials, may act as potential effectors for these reactions. Considerable evidence supports a role for calmodulin (CaM), another family of Ca^2+^ sensors with an EF hand motif that binds Ca^2+^, in modulation of SNARE-mediated exocytosis [6, 7] and endocytosis [8, 9]. Targets of CaM include multiple proteins implicated in exocytosis (e.g., Ca^2+^ channels [10], Ca^2+^/ CaM kinase II [11], rab3 [12], and Munc13 [13]), and endocytosis (e.g., calcinulin [14]). Another Ca^2+^-binding protein with an EF hand motif, parvalbumin, acts as a mobile presynaptic Ca^2+^ buffer that accelerates withdrawal of residual Ca^2+^ and decay of short-term facilitation in the calyx of held [15] and GABAergic synapses between interneurons and Purkinje cells [16]. Calretinin is upregulated in the calyx of held during postnatal development and may act as a Ca^2+^ effector or Ca^2+^ buffer to regulate transmitter release probability [17]. 

A large number of proteins are involved in presynaptic function; their synthesis, transport, and function appear to be regulated by presynaptic firing activity [18–20], Sympathetic superior cervical ganglion (SCG) neurons, which form a well-characterized cholinergic synapse in long-term culture [21, 22], are an ideal cell model [21, 23] with which to investigate these processes. The SCG neuron has a large cell body and nucleus that allows for the manipulation of gene expression and function in mature neurons *via* acute microinjection of cDNA, small interfering RNA (siRNA), dominant-negative transgenes, peptides, antibodies, and metabolites [21, 24–27], an approach not technically feasible for cultured neurons from the central nervous system. In addition, synaptic activity and short-term plasticity, as it relates to the size and replenishment of functional synaptic vesicle pools, can be accurately monitored by recording excitatory postsynaptic potentials (EPSPs) evoked by paired or repetitive action potentials in presynaptic neurons. Using this approach, we recently uncovered a critical role for CaM in presynaptic short-term plasticity [26, 28]. Our new findings concerning activity-dependent regulation of synaptic efficacy by modulating synaptic vesicle exocytosis which underlies memory formation in the brain are summarized in this paper. CaM is a Ca^2+^ effector sensing residual Ca^2+^ that mediates time- and space-dependent synaptic depression and facilitation *via* effects on Ca_V_2 channel gating [26] and vesicle replenishment in the readily releasable pool (RRP) [28]. To maintain synaptic remodeling, mitochondrial ATP biogenesis supports synaptic transmission, including efficient mobilization of synaptic vesicles into the RRP for the generation of short-term plasticity [29].

## 2. Ca_**V**_2.1 Channel Regulation and Short-Term Plasticity

### 2.1. Ca^2+^/CaM Modulates Ca**V**2.1 Channel Activity

At most fast synapses in the central nervous system, Ca_V_2.1 channels are densely clustered [30], and Ca^2+^  entered through Ca_V_2.1 channels initiates synaptic transmission [31, 32]. Ca_V_2.1 channels are Ca^2+^-dependently regulated by CaM [10, 33–36] and related to neuron-specific Ca^2+^-binding proteins, calcium-binding protein 1 and visinin-like protein-2 [37–39]. CaM and the Ca^2+^-binding proteins interact with a bipartite regulatory site in the intracellular C terminus of the *α*_1_2.1 subunit [36] called the IQ-like motif, which begins with the sequence isoleucine-methionine (IM) rather than isoleucine-glutamine (IQ); they also interact with the nearby downstream CaM-binding site. Alanine substitutions in the IQ-like domain (*α*_1_2.1_IM-AA_) blocked Ca^2+^-dependent facilitation of Ca_V_2.1 channels [34, 36], whereas Ca^2+^-dependent inactivation was blocked in channels lacking the adjacent CaM-binding domain (CBD; *α*_1_2.1_ΔCBD_) [10, 35, 36, 38, 39]. 

Ca^2+^/CaM-dependent inactivation of Ca_V_2.1 channels is dependent on global elevations of Ca^2+^ [35], which in turn are dependent on the density of Ca^2+^ channels, local Ca^2+^ buffers, the volume of the intracellular compartment, and other differences in the cellular context in which the channels are located. Ca^2+^-dependent inactivation of Ca_V_2.1 channels is observed in transfected cells overexpressing Ca_V_2.1 channels [10, 34, 35] and in the nerve terminals of the calyx of Held [40, 41], where Ca_V_2.1 channels are densely localized and large Ca^2+^ transients are generated. In contrast, Ca^2+^-dependent inactivation is not reliably observed in the large neuronal cell bodies of Purkinje neurons [42] or SCG neurons [26].

### 2.2. Ca^2+^/CaM-Mediated Ca_V_2.1 Channel Modulation, Synaptic Facilitation and Depression

Paired-pulse facilitation (PPF) and paired-pulse depression (PPD) are both mediated by Ca^2+^/CaM-dependent regulation of Ca_V_2.1 channels [26]. EPSPs recorded by pairs of action potentials with varied stimulation intervals show PPD and PPF in synaptically connected SCG neurons in which the presynaptic neuron was transfected with the *α*_1_2.1_WT_. PPD with short interstimulus interval (<40 ms) was blocked by the *α*_1_2.1_ΔCBD_ transfection, while PPF with intermediate interstimulus interval (50–100 ms) was blocked by the *α*_1_2.1_IM-AA_transfection. Thus, temporal regulation of Ca_V_2.1 channels contributes to PPD and PPF by changing the release probability in an activity-dependent manner in response to Ca^2+^ entry during the first action potential of the pair, suggesting a spatial change in Ca^2+^ concentration at the active zone and also regulates Ca_V_2.1 channel activity.

**Figure 1:**
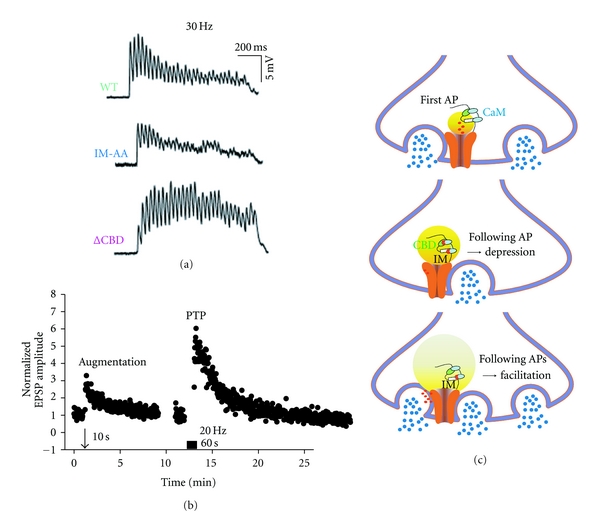
Presynaptic facilitation and depression mediated by Ca_V_2.1 channel facilitation and inactivation. (a) Averaged trace of EPSPs (*n* = 5 − 12), in which Ca_V_2.1 channels were the only active channels in the presence of *ω*-conotoxin GVIA, evoked by action potentials with 1 s train at 30 Hz. (b) Normalized amplitudes of EPSPs recorded every 2 s in the presence of *ω*-Conotoxin GVIA. Conditioning stimuli were applied at the indicated times at 20 Hz for 10 s to evoke augmentation and at 20 Hz for 60 s to induce PTP. Adapted from Mochida et al., 2008 [26]. (c) Model illustrating Ca_V_2.1-mediated mechanisms of synaptic depression, facilitation, and augmentation. In synaptic depression, CaM sensing local Ca^2+^ interacts with the CaM-binding domain (CBD) to cause channel inactivation and reduce Ca^2+^ entry, thus, reducing neurotransmitter release. In synaptic facilitation and augmentation, CaM sensing global Ca^2+^ interacts with the IQ-like motif to cause channel facilitation and increase in Ca^2+^ entry, and subsequently neurotransmitter release increases.

Neural information is encoded in bursts of synaptic activity *in vivo*. During bursts of action potentials, the CaM-dependent regulation of Ca_V_2.1 is involved in the short-term synaptic plasticity (Figure 1). Repetitive stimulation produces frequency-dependent regulation of EPSPs in SCG neurons. As the frequency of stimulation was increased, synaptic transmission mediated by the *α*_1_2.1_WT_ showed increased facilitation followed by depression during the trains of 1 s (Figure 1(a), WT). This form of short-term synaptic plasticity resembles that recorded at the calyx of Held in response to repetitive stimuli [40, 41, 43]. The facilitation is reduced by the IM-AA mutation (Figure 1(a), IM-AA) but not by the ∆CBD mutation (Figure 1(a), ∆CBD), suggesting that enhancement of neurotransmission following 1 s trains requires CaM binding to the IQ-like domain. In contrast to the facilitation followed by depression of synaptic transmission observed during trains for WT, the EPSP size increased steadily during trains (<30 Hz) for ∆CBD (Figure 1(a), ΔCBD). Facilitation of the ΔCBD mutant was greater than the WT and the IM-AA mutant throughout the train, and depression was much slower, suggesting that, similar to paired-pulse experiments, CaM-dependent inactivation of Ca_V_2.1 channels shapes the time course of short-term synaptic plasticity by determining the timing of the peak of synaptic facilitation during the train as well as the steady-state level of synaptic depression at the end of the train. 

Two forms of presynaptic short-term enhancement of synaptic transmission, termed augmentation and posttetanic potentiation (PTP), last for seconds to minutes [44]. In SCG neurons, augmentation is elicited with 10 s trains [45], and PTP is elicited with 60 s trains [46, 47]. Augmentation observed with 10 Hz, 20 Hz, and 40 Hz trains is significantly reduced by the IM-AA mutation, whereas the ΔCBD mutation has little effect on the magnitude of the augmentation [26]. Thus, Ca^2+^/CaM-dependent facilitation of Ca^2+^ entry significantly contributes to an intermediate-length form of synaptic enhancement and augmentation, which has a time course of tens to hundreds of milliseconds [26]. In contrast to augmentation, the Ca^2+^ signal that induces PTP does not require Ca^2+^/CaM-dependent facilitation. Consistent with this idea, previous reports suggested that PTP, but not augmentation, resulted from the slow efflux of mitochondrial Ca^2+^ accumulated during titanic stimulation [48] and also involved activation of protein kinase C [49, 50].

### 2.3. Ca^2+^/CaM-Mediated Ca**V**2.1 Channel Modulation and Presynaptic Short-Term Plasticity

Short-term synaptic plasticity is crucial for encoding information in the nervous system [51]. Changes in the concentration of residual Ca^2+^ are temporally sensed by a Ca^2+^ sensor CaM, resulting in Ca^2+^-dependent facilitation and inactivation of presynaptic Ca_V_2.1 currents. These currents can mediate short-term synaptic facilitation and depression, which are conserved forms of plasticity at many different types of synapses [44]. Evidently, by causing facilitation and inactivation of Ca_V_2.1 channels, residual Ca^2+^ can actually control “instantaneous” Ca^2+^ during an action potential, shape the local Ca^2+^ transient at the active zone, and cause facilitation, augmentation, and depression of synaptic transmission. The postsynaptic response is, therefore, controlled by Ca^2+^/CaM-dependent modulation of presynaptic Ca^2+^  entry, which acts to encode the information contained in the frequency of presynaptic firing of action potentials for transmission to the postsynaptic cell. Thus, presynaptic Ca^2+^ channel regulation by CaM is a major molecular mechanism underlying information processing in the nervous system.

## 3. CaM, mGluR and Munc 18-1 Interaction and Synaptic Facilitation

### 3.1. Presynaptic mGluRs

Presynaptic group III metabotropic glutamate receptors (mGluR4, mGluR7, and mGluR8) are expressed in the presynaptic active zone and reduce synaptic vesicle release upon stimulation by agonists [52–55]. A prominent enigma concerning the function of group III mGluRs is depicted in the case of mGluR4: short-term synaptic facilitation is impaired in mGluR4-KO mice although the expression of the plasticity is not reduced by pharmacological blockade of mGluR4 [52, 56]. These findings suggest the existence of some ligand-independent functions of the receptor linked to synaptic vesicle release [52]. It has been shown that Ca^2+^-dependent CaM binds to the intracellular C-terminal tail (ct) of group III mGluRs [28, 57]. Because the affinity of CaM for Ca^2+^ [58] corresponds to the required residual Ca^2+^ level for the expression of short-term facilitation, a molecule that can bind to the ct region of group III mGluRs and have an interaction with the receptor that is regulated by Ca^2+^-activated CaM is a good candidate for mGluR-mediated expression of short-term synaptic facilitation.

### 3.2. CaM and Munc18-1 Interactions with Presynaptic mGluRs

Munc18-1 is a protein essential for neurotransmission [59] and promotes SNAREs-mediated vesicle fusion [60]. Coimmunoprecipitation and *in vitro* binding experiments indicated that either mGluR4 or mGluR7 directly interacted with Munc18-1 in the brain. Group II mGluRs are also expressed presynaptically and modulate synaptic transmission [53–55]. However, group II mGluRs are localized in the preterminal portions of axons but not in the active zone [55] and do not interact with Munc18-1. Munc18-1 can be divided into three domains [61]; it binds to ct-mGluR4 through domain 1 [28]. 

CaM binds to the membrane-proximal region of group III ct-mGluRs [57, 62]. Munc18-1 binds to this region of ct-mGluR4; the interaction is disrupted by Ca^2+^-activated CaM at a concentration of residual Ca^2+^ [44]. Although Munc18-1 does not have an apparent Ca^2+^-binding motif, the interaction between ct-mGluR4 and Munc18-1 is enhanced with increasing concentrations of Ca^2+^, with saturation at 3 *μ*M. In the presence of CaM, mGluR4 binds Munc18-1 at the resting Ca^2+^ level of 0.1 *μ*M and releases it above the Ca^2+^ level of 1 *μ*M, corresponding to the residual Ca^2+^ level after an action potential.

### 3.3. mGluR4-Mediated Regulation of Basal Transmitter Release and Synaptic Facilitation

Function of the mGluR4/Munc18-1 interaction in synaptic transmission is the reduction of basal transmitter release [28]. At synapses of cultured SCG neurons, where mGluR4 is not expressed [63], the injection of mGluR4 peptide, residues 849–889, reduced gradually the amplitude of EPSPs. The time course and extent of inhibition were similar to that caused by the injection of the domain 1 of Munc18-1, suggesting that the mGluR4/Munc18-1 interaction is inhibitory to synaptic transmission.

Generally, a decrease in the initial probability of transmitter release leads to a larger synaptic enhancement or a reduction of synaptic depression [44, 64]. From the above inhibitory role of mGluR4 peptide in synaptic transmission and the operating range of the Ca^2+^-sensing mechanism, mGluR4C appears to be responsible for the expression of short-term synaptic facilitation; at the very least it reduces short-term synaptic depression. Acutely, PPD, but not PPF, was suppressed in the presence of mGluR4C peptide, suggesting that mGluR4 peptide introduces a facilitating property to these synapses which is apparent when these synapses exhibit PPD. This evidence is consistent with the idea that the absence in the ct region of mGluR4 accounts for the impaired expression of PPF in mGluR4-KO mice [56].

### 3.4. The Role of Ca^2+^/CaM, Munc18, and mGluR Interactions in Activity-Dependent Synaptic Transmission

**Figure 2:**
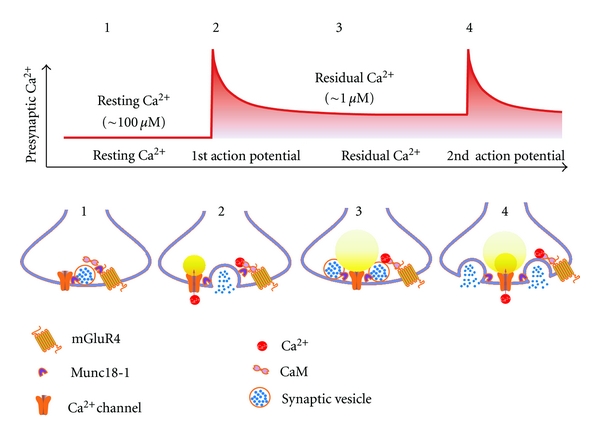
Model illustrating presynaptic short-term facilitation by Ca^2+^/CaM-mediated Munc18-1 release from mGluR4. Step  1: At the resting Ca^2+^  level, Munc18-1 is sequestered by mGluR4. Step  2: Owing to the low availability of Munc18-1 for SNARE, the initial synaptic vesicle release is small. Steps  2-3: Following the action potential, CaM sensing residual Ca^2+^ liberates Munc18-1 from mGkuR4. Step  4: The higher availability of Munc18-1 for SNARE enables a greater release of synaptic vesicles in response to the second action potential, which is derived in rapid succession. Adapted from Nakajima et al., 2009 [28].

An accumulation of evidence suggests a model for the mGluR4/Munc18-1 interaction in the regulation of synaptic transmission in the brain. When neurons are inactive, Munc18-1 is sequestered by mGluR4; therefore, basal synaptic transmission is kept low. After the action potential, residual Ca^2+^ activates CaM, which in turn liberates Munc18-1 from mGluR4, causing short-term synaptic facilitation (Figure 2). This Ca^2+^-sensing mechanism demonstrates a function for mGluR4 in the expression of short-term facilitation and explains the discrepancy between pharmacological blockade and gene targeting of mGluR4, revealed clearly at the parallel fiber synapses onto cerebellar Purkinje cells [52, 56]. Another interesting implication of our findings is that the presence of mGluR4 is responsible for the expression of certain synaptic properties (e.g., mGluR4-positive facilitating parallel fiber synapses and mGluR4-negative depressing climbing fiber synapses onto the same cerebellar Purkinje cells [44, 51, 65]). Although Munc18-1 is known to be an essential protein for synaptic transmission, evidence for both positive and negative roles of Munc18-1 in synaptic transmission has been reported [59]. If Munc18-1 has a negative role in synaptic transmission, then our data suggests that the ct-mGluR4/Munc18-1 interaction augments the inhibitory function of Munc18-1. In this case, disruption of this negative complex by Ca^2+^/CaM would relieve Munc18-1-mediated inhibition and consequently lead to synaptic facilitation. Thus, no matter whether Munc18-1 had a positive or negative role in synaptic vesicle release, the Ca^2+^-sensing system would operate in the expression of short-term synaptic facilitation.

## 4. Conclusion

Neuronal firing activity controls the functions of synaptic proteins and dynamically remodels synaptic efficacy. Ca^2+^-binding proteins that sense residual Ca^2+^, which temporally and spatially accumulates in the active zone during trains of action potentials, act as potential effectors for these reactions at the presynaptic terminal. Two pieces of evidence are shown in this article (Figures 1 and 2). First, CaM interacts with a bipartite regulatory site of the *α*_1_2.1 subunit at the mouth of Ca^2+^ channels, senses firing-dependent temporal changes in local and global elevations of Ca^2+^, and regulates activity of Ca^2+^ channels for SNAREs-mediated synaptic vesicle exocytosis [33, 66]. The CaM regulation of Ca^2+^ channels results in presynaptic depression and facilitation (Figure 1). Secondly, CaM regulates synaptic efficacy by controlling the supply of Munc 18-1 at the active zone (Figure 2). Munc18-1, an essential protein for SNAREs-mediated synaptic vesicle membrane fusion [59], binds to mGluR4 in the active zone [53–55] at the resting state. With elevation of Ca^2+^, Ca^2+^/CaM binds to mGluR4 in place of Munc18-1 [57, 62]. The Ca^2+^/CaM regulation of the mGluR4/Munc18-1 interaction results in presynaptic facilitation. The presynaptic events shown here control synaptic efficacy and encode postsynaptic activity that underlie generation of the synaptic plasticity resulting in regulation of neural circuits.

## References

[1] Südhof TC (2004). The synaptic vesicle cycle. *Annual Review of Neuroscience*.

[2] Nishiki TI, Augustine GJ (2004). Synaptotagmin I synchronizes transmitter release in mouse hippocampal neurons. *Journal of Neuroscience*.

[3] Pang ZP, Sun J, Rizo J, Maximov A, Südhof TC (2006). Genetic analysis of synaptotagmin 2 in spontaneous and Ca^2+^ -triggered neurotransmitter release. *EMBO Journal*.

[4] Sun J, Pang ZP, Qin D, Fahim AT, Adachi R, Südhof TC (2007). A dual-Ca^2+^-sensor model for neurotransmitter release in a central synapse. *Nature*.

[5] Yoshihara M, Littleton JT (2002). Synaptotagmin functions as a calcium sensor to synchronize neurotransmitter release. *Neuron*.

[6] Chen YA, Scheller RH (2001). SNARE-mediated membrane fusion. *Nature Reviews Molecular Cell Biology*.

[7] Di Giovanni J, Iborra CC, Maulet Y, Lévêque C, El Far O, Seagar M (2010). Calcium-dependent regulation of SNARE-mediated membrane fusion by calmodulin. *Journal of Biological Chemistry*.

[8] Schneggenburger R, Neher E (2000). Intracellular calcium dependence of transmitter release rates at a fast central synapse. *Nature*.

[9] Wu XS, McNeil BD, Xu J (2009). Ca^2+^ and calmodulin initiate all forms of endocytosis during depolarization at a nerve terminal. *Nature Neuroscience*.

[10] Lee A, Wong ST, Gallagher D (1999). Ca^2+^/calmodulin binds to and modulates P/Q-type calcium channels. *Nature*.

[11] Popoli M (1993). Synaptotagmin is endogenously phosphorylated by Ca^2+^/calmodulin protein kinase II in synaptic vesicles. *FEBS Letters*.

[12] Park JB, Farnsworth CC, Glomset JA (1997). Ca^2+^/calmodulin causes Rab3a to dissociate from synaptic membranes. *Journal of Biological Chemistry*.

[13] Junge HJ, Rhee JS, Jahn O (2004). Calmodulin and Munc13 form a Ca^2+^ sensor/effector complex that controls short-term synaptic plasticity. *Cell*.

[14] Marks B, McMahon HT (1998). Calcium triggers calcineurin-dependent synaptic vesicle recycling in mammalian nerve terminals. *Current Biology*.

[15] Müller M, Felmy F, Schwaller B, Schneggenburger R (2007). Parvalbumin is a mobile presynaptic Ca^2+^ buffer in the calyx of held that accelerates the decay of Ca^2+^ and short-term facilitation. *Journal of Neuroscience*.

[16] Caillard O, Moreno H, Schwaller B, Llano I, Celio MR, Marty A (2000). Role of the calcium-binding protein parvalbumin in short-term synaptic plasticity. *Proceedings of the National Academy of Sciences of the United States of America*.

[17] Felmy F, Schneggenburger R (2004). Developmental expression of the Ca^2+^-binding proteins calretinin and parvalbumin at the calyx of Held of rats and mice. *European Journal of Neuroscience*.

[18] Mochida S (2000). Protein-protein interactions in neurotransmitter release. *Neuroscience Research*.

[19] Sudhof TC (1995). The synaptic vesicle cycle: a cascade of protein-protein interactions. *Nature*.

[20] Takamori S, Holt M, Stenius K (2006). Molecular anatomy of a trafficking organelle. *Cell*.

[21] Ma H, Mochida S (2007). A cholinergic model synapse to elucidate protein function at presynatic terminals. *Neuroscience Research*.

[22] Mochida S, Nonomura Y, Kobayashi H (1994). Analysis of the mechanism for acetylcholine release at the synapse formed between rat sympathetic neurons in culture. *Microscopy Research and Technique*.

[23] Mochida S, Kobayashi H, Matsuda Y, Yuda Y, Muramoto K, Nonomura Y (1994). Myosin II is involved in transmitter release at synapses formed between rat sympathetic neurons in culture. *Neuron*.

[24] Baba T, Sakisaka T, Mochida S, Takai Y (2005). PKA-catalyzed phosphorylation of tomosyn and its implication in Ca^2+^ -dependent exocytosis of neurotransmitter. *Journal of Cell Biology*.

[25] Krapivinsky G, Mochida S, Krapivinsky L, Cibulsky SM, Clapham D (2006). The TRPM7 ion channel functions in cholinergic synaptic vesicles and affects transmitter release. *Neuron*.

[26] Mochida S, Few AP, Scheuer T, Catterall WA (2008). Regulation of presynaptic Ca_V_2.1 Channels by Ca^2+^ sensor proteins mediates short-term synaptic plasticity. *Neuron*.

[27] Mochida S, Westenbroek RE, Yokoyama CT, Itoh K, Catterall WA (2003). Subtype-selective reconstitution of synaptic transmission in sympathetic ganglion neurons by expression of exogenous calcium channels. *Proceedings of the National Academy of Sciences of the United States of America*.

[28] Nakajima Y, Mochida S, Okawa K, Nakanishi S (2009). Ca^2+^-dependent release of Munc18-1 from presynaptic mGluRs in short-term facilitation. *Proceedings of the National Academy of Sciences of the United States of America*.

[29] Ma H, Cai Q, Lu W, Sheng ZH, Mochida S (2009). KIF5B motor adaptor syntabulin maintains synaptic transmission in sympathetic neurons. *Journal of Neuroscience*.

[30] Westenbroek RE, Sakurai T, Elliott EM (1995). Immunochemical identification and subcellular distribution of the *α*(1A) subunits of brain calcium channels. *Journal of Neuroscience*.

[31] Dunlap K, Luebke JI, Turner T (1995). Exocytotic Ca^2+^ channels in mammalian central neurons. *Trends in Neurosciences*.

[32] Evans RM, Zamponi GW (2006). Presynaptic Ca^2+^ channels—integration centers for neuronal signaling pathways. *Trends in Neurosciences*.

[33] Catterall WA (2000). Structure and regulation of voltage-gated Ca^2+^ channels. *Annual Review of Cell and Developmental Biology*.

[34] DeMaria CD, Soong TW, Alseikhan BA, Alvania RS, Yue DT (2001). Calmodulin bifurcates the local Ca^2+^ signal that modulates P/Q-type Ca^2+^ channels. *Nature*.

[35] Lee A, Scheuer T, Catterall WA (2000). Ca^2+^/calmodulin-dependent facilitation and inactivation of P/Q-type Ca^2+^ channels. *Journal of Neuroscience*.

[36] Lee A, Zhou H, Scheuer T, Catterall WA (2003). Molecular determinants of Ca^2+^/calmodulin-dependent regulation of Ca_V_2.1 channels. *Proceedings of the National Academy of Sciences of the United States of America*.

[37] Few AP, Lautermilch NJ, Westenbroek RE, Scheuer T, Catterall WA (2005). Differential regulation of Ca_V_2.1 channels by calcium-binding protein 1 and visinin-like protein-2 requires N-terminal myristoylation. *Journal of Neuroscience*.

[38] Lautermilch NJ, Few AP, Scheuer T, Catterall WA (2005). Modulation of Ca_V_2.1 channels by the neuronal calcium-binding protein visinin-like protein-2. *Journal of Neuroscience*.

[39] Lee A, Westenbroek RE, Haeseleer F, Palczewski K, Scheuer T, Catterall WA (2002). Differential modulation of Ca_V_2.1 channels by calmodulin and Ca^2+^-binding protein 1. *Nature Neuroscience*.

[40] Forsythe ID, Tsujimoto T, Barnes-Davies M, Cuttle MF, Takahashi T (1998). Inactivation of presynaptic calcium current contributes to synaptic depression at a fast central synapse. *Neuron*.

[41] Xu J, Wu LG (2005). The decrease in the presynaptic calcium current is a major cause of short-term depression at a calyx-type synapse. *Neuron*.

[42] Chaudhuri D, Alseikhan BA, Chang SY, Soong TW, Yue DT (2005). Developmental activation of calmodulin-dependent facilitation of cerebellar P-type Ca^2+^ current. *Journal of Neuroscience*.

[43] Borst JGG, Sakmann B (1998). Facilitation of presynaptic calcium currents in the rat brainstem. *Journal of Physiology*.

[44] Zucker RS, Regehr WG (2002). Short-term synaptic plasticity. *Annual Review of Physiology*.

[45] Stevens CF, Wesseling JF (1999). Augmentation is a potentiation of the exocytotic process. *Neuron*.

[46] Magleby KL (1973). The effect of tetanic and post tetanic potentiation on facilitation of transmitter release at the frog neuromuscular junction. *Journal of Physiology*.

[47] Magleby KL, Zengel JE (1975). A dual effect of repetitive stimulation on post tetanic potentiation of transmitter release at the frog neuromuscular junction. *Journal of Physiology*.

[48] Tang YG, Zucker RS (1997). Mitochondrial involvement in post-tetanic potentiation of synaptic transmission. *Neuron*.

[49] Beierlein M, Fioravante D, Regehr WG (2007). Differential expression of posttetanic potentiation and retrograde signaling mediate target-dependent short-term synaptic plasticity. *Neuron*.

[50] Brager DH, Cai X, Thompson SM (2003). Activity-dependent activation of presynaptic protein kinase C mediates post-tetanic potentiation. *Nature Neuroscience*.

[51] Abbott LF, Regehr WG (2004). Synaptic computation. *Nature*.

[52] Lorez M, Humbel U, Pflimlin MC, Kew JNC (2003). Group III metabotropic glutamate receptors as autoreceptors in the cerebellar cortex. *British Journal of Pharmacology*.

[53] Nakanishi S (1992). Molecular diversity of glutamate receptors and implications for brain function. *Science*.

[54] Pin JP, Duvoisin R (1995). The metabotropic glutamate receptors: structure and functions. *Neuropharmacology*.

[55] Shigemoto R, Kinoshita A, Wada E (1997). Differential presynaptic localization of metabotropic glutamate receptor subtypes in the rat hippocampus. *Journal of Neuroscience*.

[56] Pekhletski R, Gerlai R, Overstreet LS (1996). Impaired cerebellar synaptic plasticity and motor performance in mice lacking the mGluR4 subtype of metabotropic glutamate receptor. *Journal of Neuroscience*.

[57] O’Connor V, El Far O, Bofill-Cardona E (1999). Calmodulin dependence of presynaptic metabotropic glutamate receptor signaling. *Science*.

[58] Chin D, Means AR (2000). Calmodulin: a prototypical calcium sensor. *Trends in Cell Biology*.

[59] Rizo J, Südhof TC (2002). Snares and munc18 in synaptic vesicle fusion. *Nature Reviews Neuroscience*.

[60] Shen J, Tareste DC, Paumet F, Rothman JE, Melia TJ (2007). Selective activation of cognate SNAREpins by Sec1/Munc18 proteins. *Cell*.

[61] Misura KMS, Scheller RH, Weis WI (2000). Three-dimensional structure of the neuronal-Sec1-syntaxin 1a complex. *Nature*.

[62] Nakajima Y, Yamamoto T, Nakayama T, Nakanishi S (1999). A relationship between protein kinase C phosphorylation and calmodulin binding to the metabotropic glutamate receptor subtype 7. *Journal of Biological Chemistry*.

[63] Kammermeier PJ, Ikedal SR (2002). Metabotropic glutamate receptor expression in the rat superior cervical ganglion. *Neuroscience Letters*.

[64] Atwood HL, Karunanithi S (2002). Diversification of synaptic strength: presynaptic elements. *Nature Reviews Neuroscience*.

[65] Dittman JS, Kreitzer AC, Regehr WG (2000). Interplay between facilitation, depression, and residual calcium at three presynaptic terminals. *Journal of Neuroscience*.

[66] Mochida S, Sheng Z-H, Baker C, Kobayashi H, Catterall WA (1996). Inhibition of neurotransmission by peptides containing the synaptic protein interaction site of N-type Ca2+ channels. *Neuron*.

